# Deep-Blue and Hybrid-White Organic Light Emitting Diodes Based on a Twisting Carbazole-Benzofuro[2,3-b]Pyrazine Fluorescent Emitter

**DOI:** 10.3390/molecules24020353

**Published:** 2019-01-19

**Authors:** Chen-Chao Huang, Miao-Miao Xue, Fu-Peng Wu, Yi Yuan, Liang-Sheng Liao, Man-Keung Fung

**Affiliations:** 1 Jiangsu Key Laboratory for Carbon-Based Functional Materials & Devices, Institute of Functional Nano & Soft Materials (FUNSOM), Soochow University, Suzhou 215123, China; cchuang2017@stu.suda.edu.cn (C.-C.H.); xuemiaomiao0202@yahoo.com (M.-M.X.); wufupeng520@126.com (F.-P.W.); yyuanac@126.com (Y.Y.); 2 Institute of Organic Optoelectronics, Jiangsu Industrial Technology Research Institute (JITRI), 1198 Fenhu Dadao, Wujiang, Suzhou 215123, China

**Keywords:** deep-blue fluorescence, hybrid-white organic light-emitting diode, donor-acceptor molecules, molecular twisting, bifunctional material

## Abstract

A novel deep-blue fluorescent emitter was designed and synthesized. The external quantum efficiency (η_EQE_) of the blue-emitting, doped, organic light-emitting diode (OLED) was as high as 4.34%. The device also exhibited an excellent color purity with Commission Internationale de l’Eclairage (CIE) coordinates of x = 0.15 and y = 0.05. In addition, the triplet energy had a value of 2.7 eV, which is rare for an emitter with deep-blue emission, which makes it a preferred choice for high-performance white OLEDs. By optimizing the device architectures, the color of hybrid-white OLEDs could be tunable from warm white to cool white using the aforementioned material as a bifunctional material. That is, the η_EQE_ of the hybrid warm-white OLED is 20.1% with a CIE x and y of 0.46 and 0.48 and the η_EQE_ of the hybrid cool-white OLED is 9% with a CIE x and y of 0.34 and 0.33.

## 1. Introduction

Organic light-emitting diodes (OLEDs) have been widely used as small screen displays and have been under development [1,2,3] for use in large-area displays since Tang’s group reported the first efficient OLED in 1987. There have been significant breakthroughs in the development of green and red OLEDs, including phosphorescent and fluorescent emitters [4,5,6,7,8,9,10,11,12]. However, due to the relatively poor stability of blue phosphorescent materials, blue fluorescent materials, especially deep-blue fluorescent materials, are still indispensable in OLEDs. In order to meet the demands of color saturation in full-color displays and lighting, good color purity and Commission Internationale de l’Eclairage (CIE) coordinates with a small y value of deep blue emitters are the key factors to achieving good performance full-color and white OLEDs [13,14,15]. According to the European Broadcasting Union standard and the National Television System Committee (NTSC) standard for display applications, a perfect deep-blue color requires CIE (x, y) coordinates of (0.15, 0.06) or (0.14, 0.08) as well as excellent color purity with a narrow full width at half maximum (FWHM) [16,17].

However, the fabrication of high-efficacy deep-blue OLEDs is still a big challenge, which is due to the limited choices and intrinsically wide band gap of deep-blue materials as well as poor carrier balance in blue-emitting devices [18,19,20,21,22]. Generally speaking, constructing donor-acceptor (D-A) molecules is one of the most commonly used methods to improve the charge injection and carrier transport of OLEDs. Meanwhile, a meta-linked strategy, instead of para- and ortho- strategies, is usually adopted to construct wide-band gap materials [23]. Meta-linkage could partially decrease the effect of the linkage on the conjugation length, and therefore a wide optical gap can be obtained. Recently, our group has designed and synthesized benzofuro [2,3-b]pyrazine unit (BFPz) as an acceptor moiety into a D-A system to form a novel blue fluorescent material named TPA (triphenylamine)–BFPz. We found that the molecule that used BFPz as the acceptor possesses good fluorescent and electroluminescent properties with a η_EQE_ reaching 4.42% for a sky-blue OLED [24]. However, the CIE of devices based on the abovementioned material has yet to reach the requirements of deep blue. In fact, there are a few deep-blue emitters that can give rise to devices with high η_EQE_ and excellent color purity. 

In this work, we designed and synthesized an extremely deep-blue emitting material with a CIE y coordinate of ≤0.05. To achieve good color purity, we maintained the advantages of TPA–BFPz, but we overcame its disadvantage of having too large a value of CIE y by combing the BFPz unit with a 9-phenyl-9*H*-carbazole group whose electron-donating ability is weaker than that of the triphenylamine group to reduce the interaction between the donor and acceptor. On the other hand, the meta-linkage between the donor and acceptor was further constructed so as to widen the band gap of the blue emitter. As a result, an innovative molecule named BCz–BFPz was designed and synthesized by the concise synthetic method. Its fluorescence quantum efficiency (φ_F_) is as high as 98% and 90% in toluene solution and film, respectively. More importantly, the doped device that used the new blue material displays a maximum η_EQE_ of 4.34% with a narrow FWHM of 50 nm corresponding to CIE x and y coordinates of 0.15 and 0.05. 

In addition, considering the unique advantages of the blue fluorophore’s moderate stability and the high efficacy of long wavelength phosphors, we combined the deep-blue material with a yellow phosphorescent material named Iridium (III) bis(4-phenylthieno[3,2-c]pyridinato-*N*, C2′) acetylacetonate (PO-01) to construct hybrid white-emitting OLEDs. By controlling the concentration of the phosphors and optimizing the structures of the single-EML devices, we obtained both warm and cool white OLEDs. The current efficiency (CE) and η_EQE_ of the warm white OLED are 65 cd A^−1^ and 20.1%, respectively, with a low driving voltage of 3.2 V and CIE x and y coordinates of 0.46 and 0.48. The maximum efficiencies of cool white OLED are 24 cd A^−1^ and 9% with CIE x and y coordinates of 0.34 and 0.33, which is quite close to those of standard white OLEDs which have CIE x and y coordinates of 0.33 and 0.33. 

## 2. Results and Discussion

**Scheme 1:**
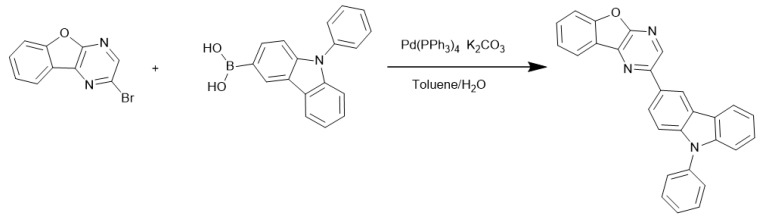
Synthetic route of BCz–BFPz.

The BCz–BFPz was synthesized in a succinct way. The synthetic route for the preparation of BCz–BFPz is shown in Scheme 1 and the detailed synthetic methods are shown in Scheme S1. Firstly, 2-bromobenzofuro[2,3-b]pyrazine (2-*Br*-BFPz) was synthesized according to the literature [24]. The BCz–BFPz was then prepared by Suzuki coupling reaction of 2-*Br*-BFPz with (9-phenyl-9*H*-carbazol-3-yl)boronic acid. The synthetic yield of BCz–BFPz was 80%. The detailed synthetic procedures are depicted in the Supplementary Materials. Their structures were fully characterized by ^1^H-NMR, ^13^C-NMR, and mass spectrometry (MS).

**Figure 1:**
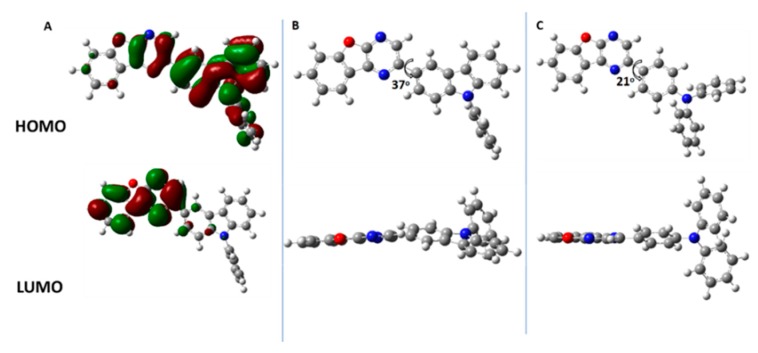
(**A**) Highest occupied molecular orbital (HOMO)/lowest unoccupied molecular orbital (LUMO) spatial distribution of BCz–BFPz; (**B**) top-view and side-view of BCz–BFPz optimized structure; (**C**) top-view and side-view of TPA–BFPz optimized structure.

Next, DFT calculation (B3LYP/6-31 g*) of the BCz–BFPz molecule was employed to gain a deeper understanding of the molecule. As shown in Figure 1, except the highest occupied molecular orbital (HOMO) and the lowest unoccupied molecular orbital (LUMO) that partly overlaps in the pyrazine part of the BFPz unit, the HOMO of BCz–BFPz is mostly located in the BCz group and the majority of LUMO is distributed in the BFPz moiety, which is similar to that of the TPA–BFPz. However, the dihedral angle between the D and A parts is larger than that of the TPA–BFPz with a more distorted angle (θ_D–A_) of 37°, which implies that the more distorted structure of BCz–BFPz arisen from the meta-linkage can lead to the blue-shifted emission. 

**Figure 2:**
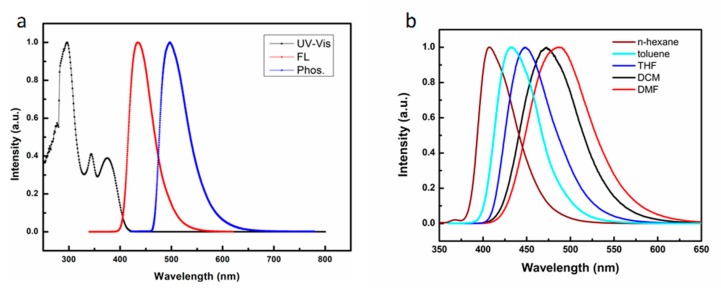
(**a**) UV-visible and fluorescence (FL) spectra of BCz–BFPz in toluene (10^−5^ mol L^−1^) and phosphorescence spectrum of BCz–BFPz in 2-methylfuran solvent at 77 K; (**b**) FL spectra of BCz–BFPz in different solvents (10^−5^ mol L^−1^).

In order to further investigate the photophysical properties, UV-visible spectrum and fluorescence (FL) spectrum of BCZ–BFPz were measured in toluene solution (10^−5^ mol L^−1^). The phosphorescence (phos.) spectrum (obtained at 77 K) of BCz–BFPz was tested in 2-methylfuran solvent. As shown in Figure 2a, three pronounced absorption peaks can be seen. The short wavelength absorption peaks at 298 nm and 344 nm can be ascribed to the π–π* transition and n–π* transition of the carbazole and BFPz center. The peak at 375 nm could be attributed to the charge transfer (CT) transition from donor (BCz) to acceptor (BFPz). Shown in the FL spectrum, the width of the emission is quite narrow with a peak at 434 nm. In Figure 2b, the triplet energy of BCz–BFPz as calculated from the onset of phos. spectrum at 77 K is 2.70 eV. Besides, the φ_F_ of BCz–BFPz was measured. Its fluorescence quantum efficiencies are as high as 98% and 90% in toluene solution and in film, respectively, which suggest that BCZ–BFPz can be an excellent deep-blue emitter for OLEDs. The FL spectra of BCZ–BFPz in different solvents were measured. As shown in Figure 2b, there is a wide red shift upon augmenting the solvent polarity, e.g., 407 nm in n-hexane and 485 nm in DMF, which may indicate the CT state of the molecule. The thermal properties of BCz–BFPz were measured by thermogravimetric analysis (TGA) (Figure S1 in the Supplementary Materials). The BCz–BFPz is thermally stable with a 5% weight-loss temperature (T_d_) of up to 371 °C. Differential scanning calorimetry (DSC) was also carried out. It was found that, as shown in the inset of Figure S1 in the Supplementary Materials, the glass transition temperature (T_g_) of BCz–BFPz is ~88 °C, which is higher than that of the commercial host (e.g., 1,3-bis(cabazol-9-yl) benzene (mCP), T_g_ ≤ 60 °C). We also performed AFM to measure the morphological stability of our film. As shown in Figure S2 in the Supplementary Materials, the root mean square roughness of the pristine BCz–BFPz was 0.955 nm. After annealing it at 80 °C for 2 h, the roughness decreased to 0.722 nm, and the film morphology did not change adversely. The electrochemical behavior of BCz–BFPz was investigated by cyclic voltammetry (CV), in which the measurement details can be found in our previous works [25,26]. As shown in Figure S3 in the Supplementary Materials, the HOMO was calculated to be −5.58 eV. The bandgap was estimated to be 3.05 eV according to the UV-visible spectrum in Figure 2a, and the LUMO was estimated to be −2.53 eV. A summary of the photophysical properties, thermal properties, and HOMO/LUMO energy levels of BCz–BFPz is shown in Table 1.

**Figure 3:**
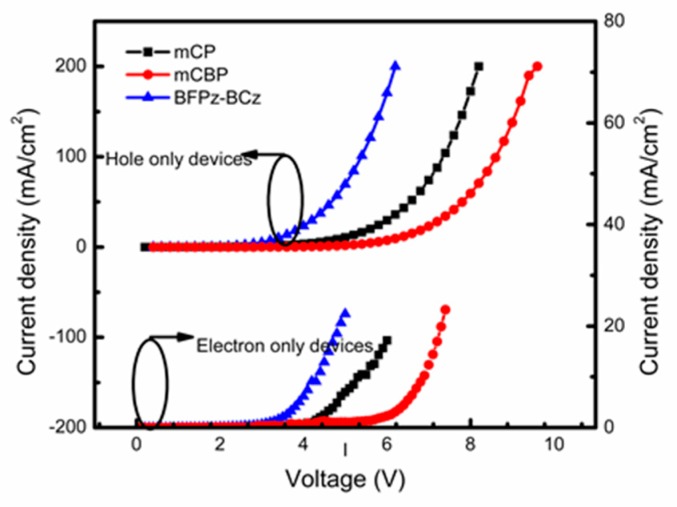
Current density versus voltage characteristics of the hole-only and electron-only devices.

To investigate the electroluminescent properties of BCz–BFPz, OLEDs with various device configurations were fabricated. The structure of the devices is as follows: ITO/HAT-CN (10 nm)/TAPC (40 nm)/TCTA (10 nm)/Host: BCz–BFPz (Y wt%, 20 nm)/TmPyPB (45 nm)/Liq (2 nm)/Al (120 nm), in which Host represents the host material 3,3-di(9H-carbazol-9-yl)biphenyl (mCBP) or mCP, and Y represents the doping concentration of BCz–BFPz in the host material. The HAT–CN and Liq represent 1,4,5,8,9,11hexaazatriphenylenehexacarbonitrile and 8-hydroxyquinolinolatolithium, which were used as hole injection and electron injection layers, respectively. The TAPC and TmPyPB, namely di-[4-(*N*,*N*-ditolyl-amino)phenyl]cyclohexane and 1,3,5-tri[(3-pyridyl)phen-3-yl]benzene were used as hole transporting and electron transporting layers, respectively. The TCTA, namely 4,4′,4-tri(*N*-carbazolyl)triphenylamine, was used as an exciton blocking layer. Another device without the use of host (non-doped device) was also fabricated for comparison. As exhibited in Table 2 and Figure S4, the driving voltages of all devices at 0.2 mA cm^−2^ were under 4 V. Compared with the doped devices, the driving voltage of the non-doped device at 0.2 mA cm^−2^ was generally lower, which suggests that BCz–BFPz may have good electron and hole transporting abilities thanks to its D-A structure. To confirm this, we measured the the current density versus voltage characteristics of hole-only and electron-only devices for comparison. The structures of the hole-only and electron-only devices are: ITO/MoO_3_ (10 nm)/TAPC (10 nm)/Z (30 nm)/TAPC (10 nm)/MoO_3_ (10 nm)/Al (120 nm) and ITO/TmPyPB (10 nm)/Z (30 nm)/TmPyPB (10 nm)/Liq (2 nm)/Al (120 nm), respectively, where Z is BCz–BFPz, mCP or mCBP. It can be deduced in Figure 3 that BCz–BFPZ has the highest hole and electron mobility compared with mCP and mCBP. 

**Figure 4:**
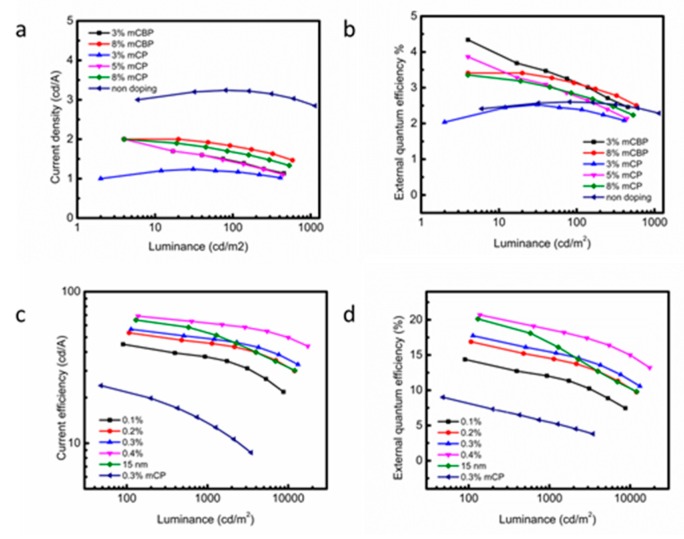
(**a**–**b**) CE-l and η_EQE_-l curves of deep-blue devices; (**c**–**d**) CE-l and η_EQE_-l curves of white devices.

**Figure 5:**
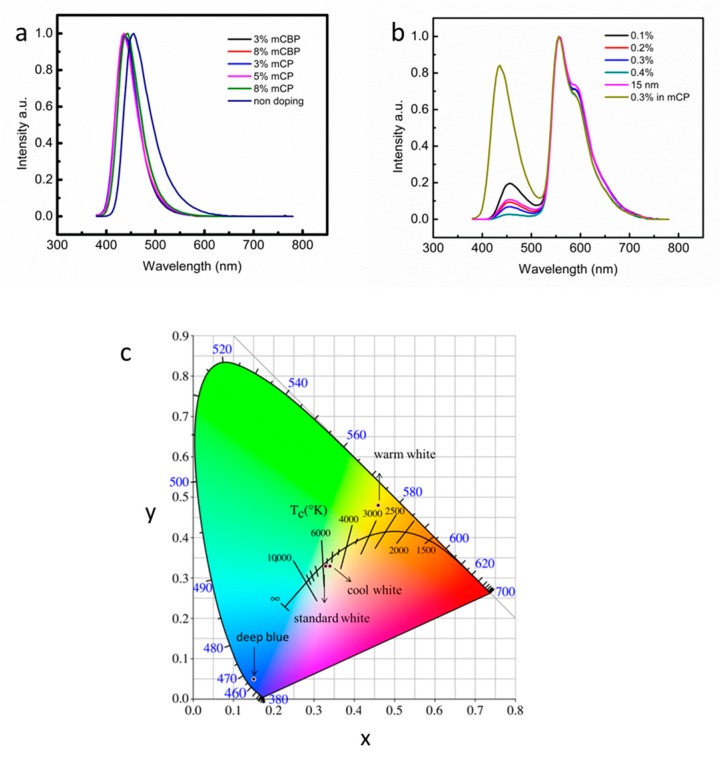
(**a**) EL spectra of deep-blue devices; (**b**) EL spectra of various white-emitting OLEDs; (**c**) the chromaticity coordinates of deep blue and white devices.

When the doping concentration of BCz–BFPz in host was kept the same, the performances of the devices (Figure 4a–b and Table 2) that were based on the mCBP/BCz–BFPz were better than that of devices based on mCP/BCz–BFPz, which may be attributed to the more efficient energy transfer from mCBP to BCz–BFPz compared with mCP. On the other hand, as displayed in Figure 5a, the electroluminescent (EL) spectrum gradually red shifted with the increase in BCz–BFPz-doping concentration. As a result, the device which used a doping concentration of 3% BCz–BFPz with mCBP as the emitting layer had the best performance with a maximum CE and η_EQE_ of 2.0 cd A^−1^ and 4.34%, respectively, at 436 nm with a narrow FWHM of 50 nm corresponding to CIE x and y coordinates of 0.15 and 0.05. 

Considering the high triplet energy of BCz–BFPz and promising application of white OLEDs, we combined the deep-blue material with a yellow phosphorescent material named iridium (III) bis(4-phenylthieno[3,2-c]pyridinato-*N*,C2′)acetyla-cetonate (PO-01) to form hybrid white OLEDs (WOLEDs) without using an interlayer, in which BCz–BFPz serves as the host material as well as blue fluorophore [27,28]. More importantly, we can obtain both warm WOLEDs and cool WOLEDs by optimizing the OLED structure. First, devices with this configuration (ITO/HAT-CN (10 nm)/TAPC (40 nm)/TCTA (10 nm)/BCz–BFPz: PO-01 (Y wt%, 20 nm)/TmPyPB (45 nm)/Liq (2 nm)/Al (120 nm)) were fabricated, in which Y represents the doping concentration of PO-01 in BCz–BFPz. As shown in Figure 4c,d, Figure S5, and Table 2, while the current efficiency increases with an increase in the doping concentration of PO-01 in BCz–BFPz, the color of the emission of the devices becomes more and more warm white. When the doping concentration of PO-01 in BCz–BFPz reached 0.4%, the devices had the highest efficacy with a maximum η_EQE_, power efficiency (PE), and CE of 20.7%, 69.2 lm W^−1^, and 69 cd A^−1^, respectively, with a turn-on voltage of 3.1 V (at a current density of 0.2 mA cm^−2^). At a brightness of 1000 cd/m^2^, the device efficiencies were also maintained at 18.5% and 62 cd/A.

However, as seen in Figure 5b, the blue emission of this device seems too low. Hence, in order to balance the device performance and the color purity of white OLEDs, we fine-tuned the OLED structure with a configuration of ITO/HAT-CN(10 nm)/TAPC (40 nm)/TCTA (10 nm)/BCz–BFPz: PO-01 (0.4 wt%, 20 nm)/BCz–BFPz (15 nm)/TmPyPB (45 nm)/Liq (2 nm)/Al. As a result, we ended up with a new device that satisfies the requirement of color without sacrificing efficacy with a maximum η_EQE_, PE, and CE of 20.1%, 63.5 lm W^−1^, and 65 cd A^−1^, respectively, with CIE x and y coordinates of 0.46 and 0.48, corresponding to a color rendering index (CRI) of 41 at 10 mA/cm^2^. The device exhibited an EQE and current efficiency of 16.7% and 53.5 cd/A at a brightness of 1000 cd/m^2^. In addition to the warm-white emission, cool white OLED also plays an important role in application and daily life. However, there are only occasional reports in previous research where the CIE x and y coordinates were close to 0.33 and 0.33 [29,30,31]. In order to satisfy the requirements of cool white, we optimized the OLED structure with a configuration of ITO/HAT-CN (10 nm)/TAPC (40 nm)/TCTA (10 nm)/mCP: BCz-BFPz: PO-01 (5 wt%, 0.3 wt%, 20 nm)/TmPyPB (45 nm)/Liq (2 nm)/Al. As shown in Table 2, Figure 4c–d, and Figure 5c, although the efficiencies of the cool-white device is lower than that of the aforementioned warm-white devices, the CIE is quite close to the standard white. That is, the maximum CE and η_EQE_ of the device are 24 cd A^−1^ and 9%, respectively, with CIE x and y coordinates of 0.34 and 0.33 and a CRI of 54 measured at 10 mA/cm^2^, which indicate that we have not only acquired high-performance deep-blue OLEDs but also realized the control of hybrid OLEDs from warm white to cool white by optimizing the device structure. The EL spectra of the aforementioned warm white and cool white OLEDs as a function of current density are shown in Figure S6.

## 3. Conclusions

In summary, by controlling the interaction and connection type between the donor and acceptor, we have designed and synthesized a new deep-blue material, BCz–BFPz. The doped device based on BCz–BFPz possesses an excellent performance as the η_EQE_ is up to 4.34% corresponding to CIE x and y coordinates of 0.15 and 0.05. In addition, high triplet energy with the value of 2.7 eV is quite rare for a deep-blue emitter (CIE y ≤ 0.08). By designing and optimizing the structure of devices, we have demonstrated hybrid warm-white and cool-white OLEDs. The η_EQE_ and power efficiency of the hybrid warm-white OLED are as high as 20.1% and 63.5 lm W^−1^ and the CIE of the cool hybrid WOLED is 0.34 and 0.33, which are quite close to those of standard white.

## Figures and Tables

**Table 1:** Photophysical, thermal properties, and HOMO/LUMO energy levels of BCz–BFPz.

	Abs ^a^	PL ^b^	T_d_^c^	Eg ^d^	Et ^e^	HOMO ^f^	LUMO ^g^
	[nm]	[nm]	[oC]	[eV]	[eV]	[eV]	[eV]
BCz–BFPz	298, 344, 375	434	371	3.05	2.70	−5.58	−2.53

^a^ Measured in toluene solution at room temperature. ^b^ Measured in toluene solution at room temperature. ^c^ T_d_: Decomposition temperature. ^d^ E_g_: Band gap energy was calculated from the corresponding absorption onset in toluene solution. ^e^ E_t_: The triplet energy was calculated from the onset of phosphorescence spectrum at 77 K. ^f^ HOMO energy level was calculated from CV data. ^g^ LUMO energy level was calculated from the HOMO and E_g_.

**Table 2:** Summary of electroluminescence data for organic light-emitting diodes (OLEDs).

	Devices ^a^	Voltage ^d^ (V)	CE_max_^e^ (cd/A)	η_EQE max_^e^ (%)	CIE ^f^	Emission peak ^e^	FWHM ^e^
Blue ^a^	3% in mCBP	3.9	2.0	4.34	0.15, 0.050	436	50
	8% in mCBP	3.8	2.0	3.41	0.15, 0.060	444	51
	3% in mCP	3.8	1.24	2.53	0.15, 0.054	436	51
	5% in mCP	3.9	2.0	3.87	0.15, 0.057	436	51
	8% in mCP	3.7	2.0	3.35	0.15, 0.064	444	54
	non-doped	3.5	3.24	2.6	0.15, 0.148	456	61
White ^b^	0.1%	3.2	45	14.4	0.44, 0.46		
	0.2%	3.2	53.5	16.9	0.46, 0.48		
	0.3%	3.2	56.5	17.7	0.46, 0.49		
	0.4%	3.1	69	20.7	0.47, 0.51		
	0.4% and 15 nm	3.2	65	20.1	0.46, 0.48		
	0.3% and 5% in mCP ^c^	3.8	24	9.0	0.34, 0.33		

^a^ Doping concentration of BCz–BFPz in hosts; ^b^ Doping concentration of PO-01 in BCz–BFPz; ^c^ 0.3% PO-01 and 5% BCz–BFPz in mCP; ^d^ Driving voltage (at 0.2 mA cm^−2^); ^e^ CE_max_, ƞ_EQEmax_ = maximum current efficiency, external quantum efficiency; ^f^ Measured at 10 mA cm^−2^.

## Supplementary Materials

The following are available online, including Experimental section, Scheme S1. Synthetic routes of BCz–BFPz; Figure S1. TGA and DSC (the inset) curves of BCz–BFPz; Figure S2. AFM images showing (a) pristine film of BCz–BFPz; (b) BCz–BFPz annealed at 80 °C for 2 hours; Figure S3. Cyclic voltammetry curve of BCz–BFPz; Figure S4. J-V-L characteristics of deep-blue OLEDs; Figure S5. J-V-L characteristics of white OLEDs; Figure S6. EL spectra of white OLEDs with a doping concentration of 0.4% PO-01 in BCz–BFPz (a); 0.3% PO-01 and 5% BCz–BFPz in mCP (b).
