# Supplementary material for: Deep-Blue and Hybrid-White Organic Light Emitting Diodes Based on a Twisting Carbazole-Benzofuro[2,3-b]Pyrazine Fluorescent Emitter
Source: Molecules. 2019 Jan 19;24(2):353. doi: 10.3390/molecules24020353 (PMC6358809; doi:10.3390/molecules24020353)
Supplement: Supplementary file 1 [file molecules-24-00353-s001.pdf]

Deep blue and color-tunable hybrid white organic light emitting diodes based on a twisting carbazole-benzofuro[2,3-b]pyrazine fluorescent emitter

Chen-Chao Huang [a], Miao-Miao Xue [a], Fu-Peng Wu [a], Yi Yuan [a], Liang-Sheng Liao [*a,b], Man-Keung Fung [*a,b]

[a] Jiangsu Key Laboratory for Carbon-Based Functional Materials & Devices, Institute of Functional Nano & Soft Materials (FUNSOM), Soochow University, 199 Ren'ai Road, Suzhou, 215123, Jiangsu, PR China.

[b] Institute of Organic Optoelectronics, Jiangsu Industrial Technology Research Institute (JITRI), 1198 Fenhu Dadao, Wujiang, Suzhou, Jiangsu, P.R. China.

Email: lsliao@suda.edu.cn; mkfung@suda.edu.cn.

EXPERIMENTAL SECTION

Characterization

^1H NMR and ^{13}C NMR spectra were recorded by using a Varian Unity Inova 400 spectrometer at room temperature. Mass spectrum was measured by a Thermo ISQ mass spectrometer equipped with a direct exposure probe. UV-Vis absorption spectrum was obtained by a Perkin Elmer Lambda 750 spectrophotometer. PL spectrum was measured by a Hitachi F-4600 fluorescence spectrophotometer. TGA was performed with a TA SDT 2960 instrument at a heating rate of 10 ^oC/minute from 30 ^oC to 600 ^oC under nitrogen. Temperature at a 5% weight loss was used as the decomposition temperature (Td). The differential scanning calorimetry (DSC) was carried out on a TA DSC 2010 unit at a heating rate of 10 ^0C min^{-1} under nitrogen. CV was carried out using a CHI600 voltammetric analyzer at room temperature with ferrocenium-ferrocene (Fc+/Fc) as the internal standard. Dichloromethane was used as a solvent for oxidation scan with tetrabutylammonium hexafluorophosphate (TBAPF6) (0.1 M) as the supporting electrolyte. The cyclic voltammograms were obtained at a scan rate of 0.1 V s^{-1}. Surface morphology was measured by atomic force microscopy (Cypher ES).

Computational methodology

The geometrical and electronic properties of BCz-BFPz were performed by the Gaussian 09 program package. The molecular structure was optimized by b3lyp (Becke three parameters hybrid functional with Lee-Yang-Perdew correlation functionals) with the 6-31G(d) atomic basis set. Molecular orbitals were visualized by Gaussview.

Device fabrication and measurements

All OLEDs were fabricated with vacuum deposition (Suzhou Fangsheng FS-450) at a base pressure of 2´10^{-6} Torr. Commercially available ITO-coated glass with a sheet resistance of ca. 30 Ω per square was used as substrates. The ITO surface was cleaned sequentially with acetone, ethanol, and deionizer water, dried in an oven, and exposed to UV-ozone for 20 minutes. HAT-CN and Liq were deposited at a rate of 0.2-0.3 Å/s, and other organic layers were deposited at 2-3 Å/s, finally the Al electrode was deposited (ca. 5 Å/s) through a shadow mask without breaking the vacuum. All devices have an emitting area of 0.09 cm^2. The EL spectra, CIE coordinates and J-V curves of the devices were measured with a Photo Research Spectra Scan PR 655 photometer and a Keithley 2400 Source Meter constant current source at room temperature. The EQE values were calculated based on the above experimental data.

Preparation of compounds

All raw materials and reagents were purchased from commercial sources and used as received

without further purification. Solvents for chemical synthesis were purified according to the standard procedures.

Synthesis of 2-(9-phenyl-9H-carbazol-3-yl)benzofuro[2,3-b]pyrazine (BCz-BFPz)

2-bromobenzofuro[2,3-b]pyrazine (2-Br-BFPz) was prepared according to the literature.[21] A mixture of 2-bromobenzofuro[2,3-b]pyrazine (2-Br-BFPz, 1.2 g, 4.84 mmol), (9-phenyl-9H-carbazol-3-yl)boronic acid (2.69 g, 7.26 mmol), potassium phosphate tribasic (3.0 g, 14.5 mmol), tetrakis(triphenylphosphine)palladium(0) (0.3 g, 1.6 mmol), toluene (120 ml) and distilled water (12 ml) was refluxed under N_2 for 48 hours. Then reaction mixture was cooled down to room temperature, diluted with 50 mL water and extracted with ethyl acetate. The organic extracts were dried over anhydrous magnesium sulfate, filtered, and evaporated. The crude material was purified by column chromatography on silica gel using dichloromethane/n-hexane as eluent. Additional purification by sublimation resulted in 1.5 g of pure blue-green compound (BCz-BFPz).
Yield 75%; $C_{28}H_{17}N_3O$; MS (EI) m/z 411.27 $[(M+H)^+]$.^{1}H NMR (400 MHz, $CDCl_3$, δ); 8.97 (s, 1H), 8.94 (s, 1H), 8.44 (d, 1H J=8.0), 8.35 (d, 1H, J=8.0), 8.22 (d, 1H, J=8.0), 7.71-7.66 (m, 6H), 7.61-7.49 (m, 5H), 7.41 (m, 1H); ^{13}C NMR (400 MHz, $CDCl_3$, δ); 156.31, 155.74, 150.13, 141.07, 136.93, 136.89, 136.66, 130.01, 129.52, 128.44, 127.28, 126.64, 125.96, 124.68, 123.69, 123.64, 122.99, 121.79, 121.58, 120.18, 119.93, 118.87, 112.24, 109.88, 109.59.

Scheme S1. Synthetic routes of BCz-BFPz.

Figure S1. TGA and DSC (the inset) curves of BCz-BFPz.

Figure S2. AFM images showing (a) pristine film of BCz-BFPz; (b) BCz-BFPz annealed at 80 °C for 2 hours.

Figure S3. Cyclic voltammetry curve of BCz-BFPz.

Figure S4. J-V-L characteristics of deep blue OLEDs.

Figure S5. J-V-L characteristics of white OLEDs.

Figure S6. EL spectra of white OLEDs with a doping concentration of 0.4% PO-01 in BCz-BFPz (left); 0.3% PO-01 and 5% BCz-BFPz in mCP (right).

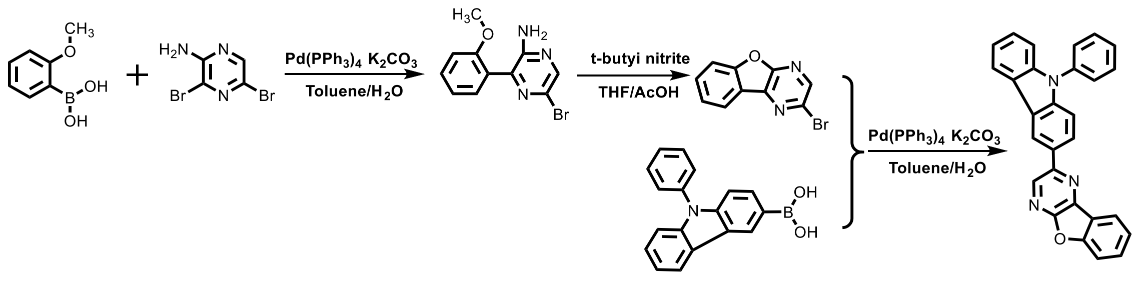

Scheme S1. Synthetic routes of BCz-BFPz.

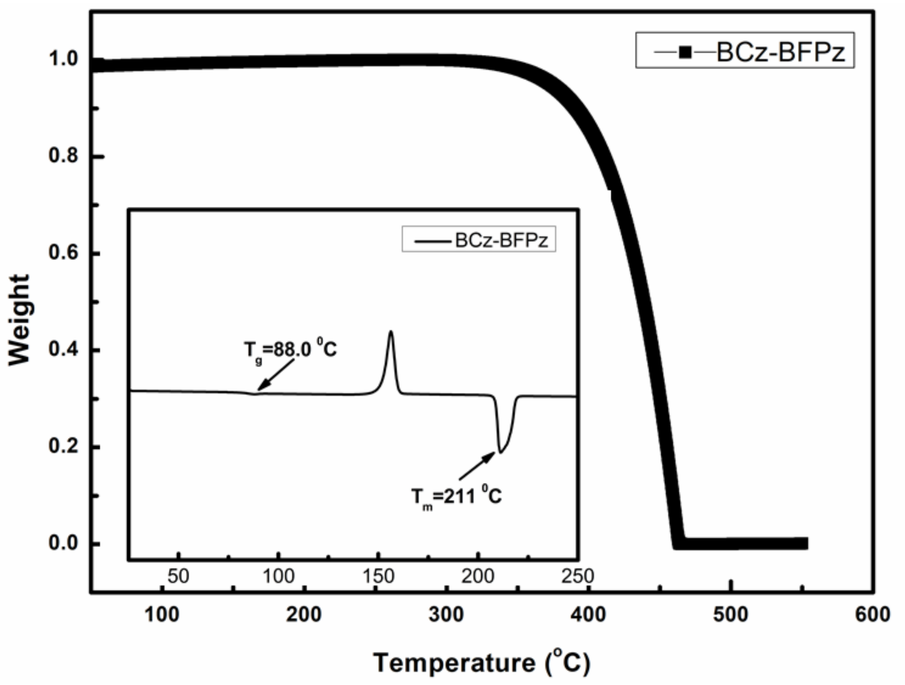

Figure S1. TGA and DSC (the inset) curves of BCz-BFPz.

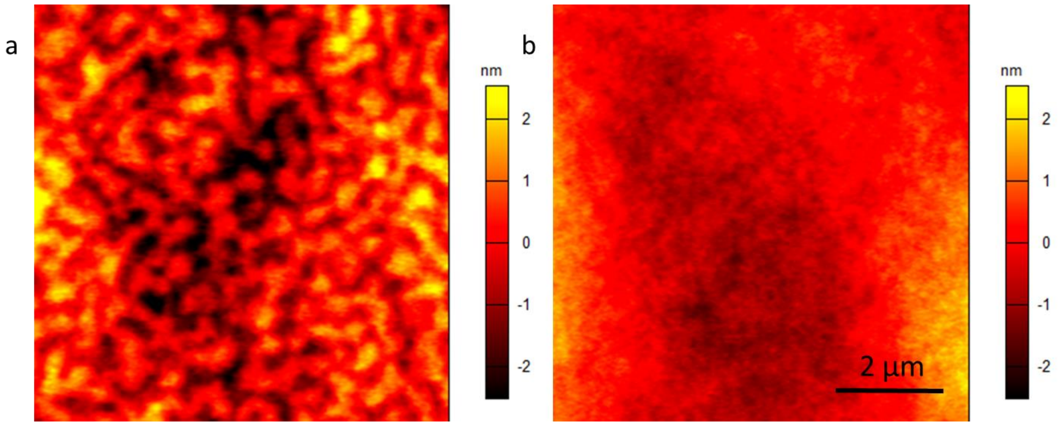

Figure S2. AFM images showing (a) pristine film of BCz-BFPz; (b) BCz-BFPz annealed at 80 °C for 2 hours.

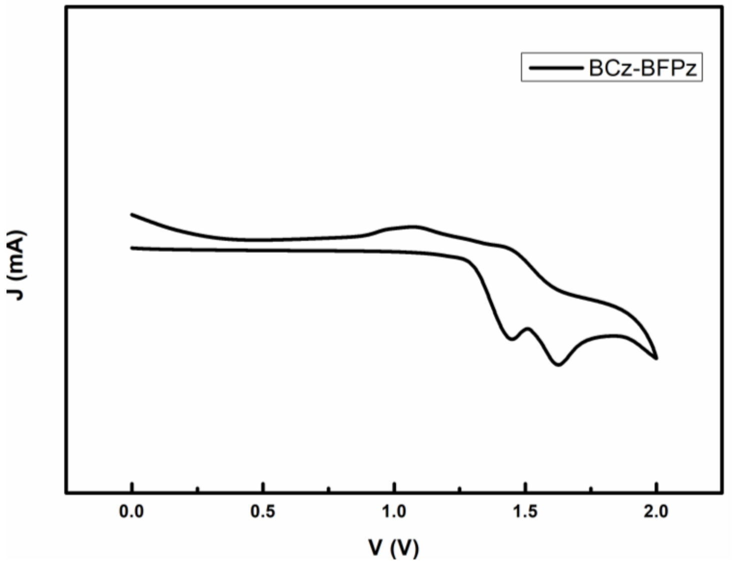

Figure S3. Cyclic voltammetry curve of BCz-BFPz.

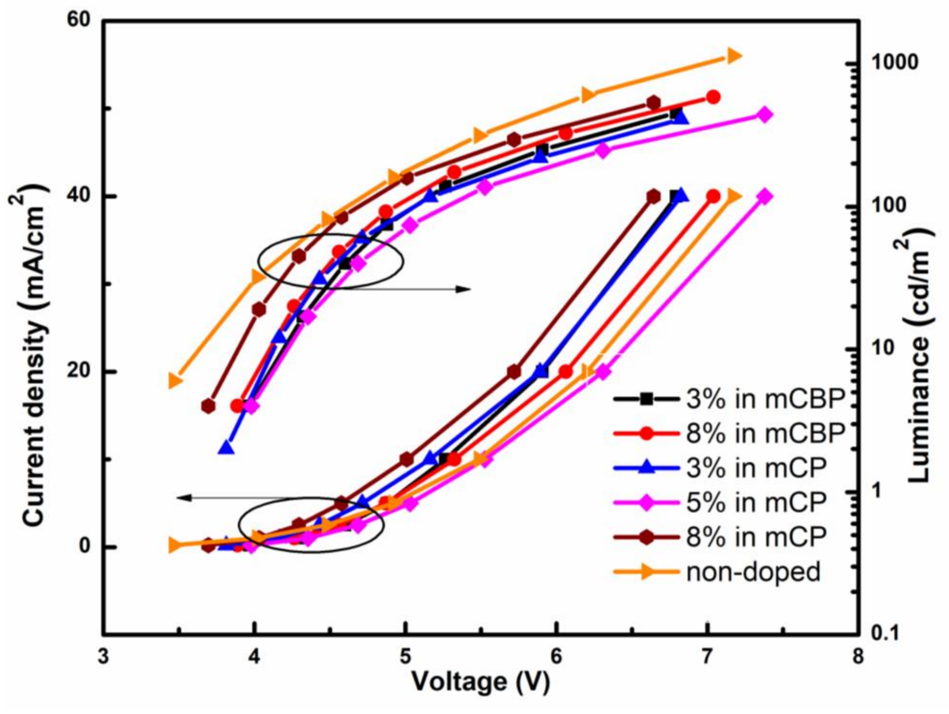

Figure S4. J-V-L characteristics of deep blue OLEDs.

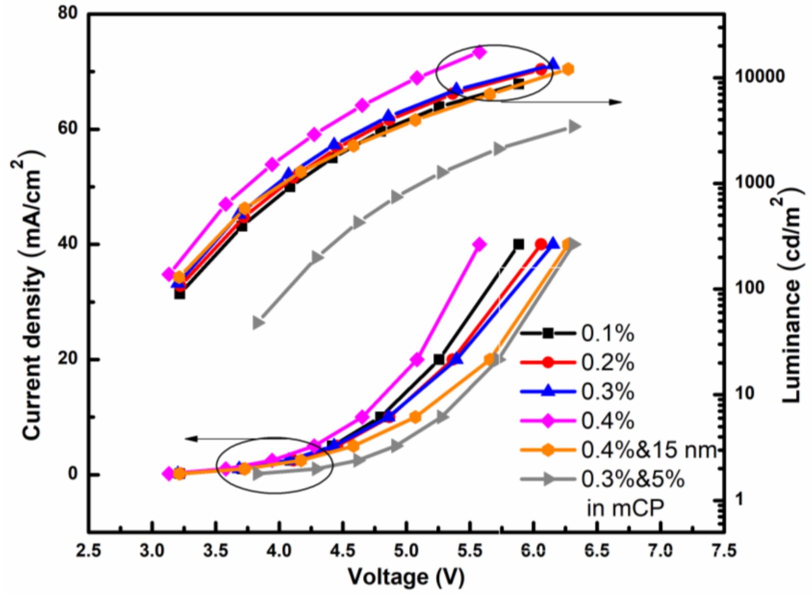

Figure S5. J-V-L characteristic of white OLEDs.

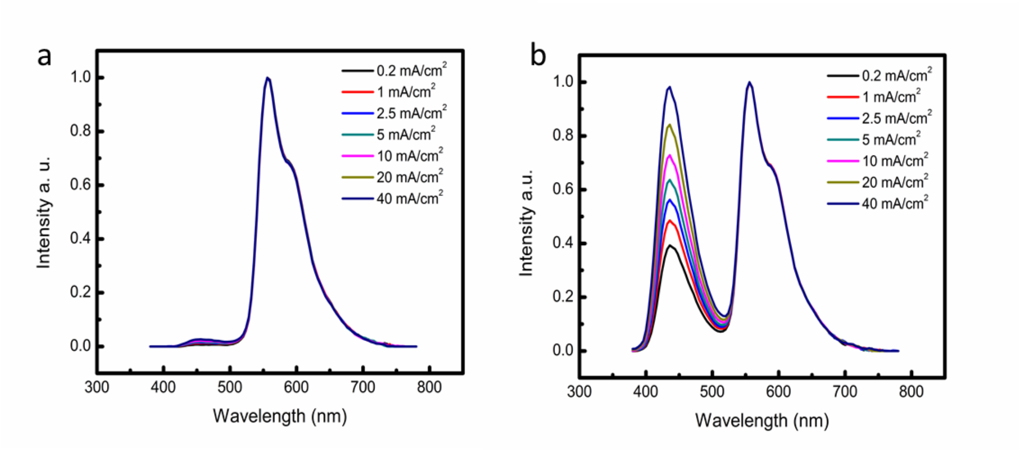

Figure S6. EL spectra of white OLEDs with a doping concentration of 0.4% PO-01 in BCz-BFPz (left); 0.3% PO-01 and 5% BCz-BFPz in mCP (right)
